# Translation and validation of the Canadian assessment of physical literacy-2 in a Danish sample

**DOI:** 10.1186/s12889-021-12301-7

**Published:** 2021-12-09

**Authors:** Peter Elsborg, Paulina S. Melby, Mette Kurtzhals, Mark S. Tremblay, Glen Nielsen, Peter Bentsen

**Affiliations:** 1 Steno Diabetes Center Copenhagen, Health Promotion Research, Copenhagen, Denmark; 2 Center for Clinical Research and Prevention, Copenhagen University Hospital – Bispebjerg and Frederiksberg, Frederiksberg, Denmark; 3 Department of Nutrition, Exercise and Sports, University of Copenhagen, Nyborg, Denmark; 4 Danish School Sports, Copenhagen, Denmark; 5 Children’s Hospital of Eastern Ontario Research Institute, Ottawa, Canada; 6 Faculty of Medicine, Department of Pediatrics, University of Ottawa, Ottawa, Canada; 7 Department of Geosciences and Natural Resource Management, University of Copenhagen, Copenhagen, Denmark

**Keywords:** Assessment, Measurement, Children, School, Physical education

## Abstract

**Background:**

The aim of this study was to translate the Canadian Assessment of Physical Literacy, second edition (CAPL-2) into Danish language, adapt it to Danish context and to test the measurement properties on a sample of Danish school children.

**Methods:**

The CAPL-2 measurement tool was translated into Danish language and adapted for the Danish context. This Danish version of the CAPL-2 was then tested on 891 Danish school children from 50 classes in 12 different schools.

**Results:**

Confirmatory factor analysis using the four-factor model, as suggested by the CAPL-2 original developers, showed an acceptable model fit for the Danish version (CFI = .973; TLI = .957; RMSEA = 0.040 (90% CI 0.033–0.054); SRMR = 0.040). Positive significant correlations between the domains were found. The domains as well as the total CAPL-2 score were found to be positively associated with physical education teachers’ assessment of their pupil’s in four central aspects of PL (i.e. enjoyment, confidence, motor skills, and diversity) indicating predictive validity. High internal consistency of the instrument used to measure motivation and confidence domain were found.

**Conclusion:**

The translated and context-adapted Danish version of CAPL-2 is a valid and reliable measurement tool ready to use in Danish research studies.

## Background

In Denmark, as across the rest of Europe, many children and young people are not meeting physical activity (PA) guidelines [1]. Recently, a longitudinal large scale representative study based on device measured PA from 8186 children and young people in Norway concluded that strategies and programs aiming to increase children and young people’s PA have been inadequate [2]. Another recent study concluded that: *“Health is not a motivating factor for adolescents; therefore, interventions designed solely to improve health are unlikely to engage them”* [3]. However, like with health, the promotion of PA alone does not provide sufficient motivation to result in behavior change, it is not a trait and it is very context dependent. This might explain why several attempts in Denmark to improve adherence to PA guidelines for children, including initiatives such as a school reform requiring 45 min of structured PA during school time have not resulted in the needed increase of daily PA among children [4]. Accordingly, systematic reviews show that school-based interventions aiming to increase PA shows small or non-significant effects and no long-term intervention effects [5, 6]. This suggests that interventions need to do more than simply inform the public of the health benefits of PA and focus on the quantity of children’s PA behavior.

One solution is for strategies and programs to stimulate development of children’s prerequisites and capacities for PA participation as a precursor to increasing PA behavior itself. Such prerequisites include PA-related competences, motivation, confidence, knowledge, and understanding. In recent years, the construct of physical literacy (PL) [7], which is an umbrella term encompassing these aspects of PA, has gained increasing attention within the research community as well as in practice, especially in Australia, Canada and the UK [8]. Although slightly varying definitions exist, it has been argued both theoretically [7, 9] and empirically [10–14] that PL is in fact a prerequisite of PA participation, making it foundational for the development, design and evaluation of PA interventions. Studies have also shown a direct association between PL and children’s health [15, 16] and how other behavioral habits, such as screen time, hinder PL development [17]. However, in order to evaluate the effectiveness of interventions that target an increase in children’s PL, there is a need for valid measures of PL. If valid measures of PL are implemented systematically nationally or internationally it will not only enable researchers, but also funders, policymakers, and practitioners to understand what projects, pedagogies and policies are most effective in promoting children’s PL [18]. However, no such measure exists in Denmark. Recently, a systematic review of measures of overall PL and it’s individual subdomains for children aged 7–12 years concluded that amongst the reviewed measurement tools, the Canadian Assessment of Physical Literacy, second edition (CAPL-2) was one of two measures of PL with strong evidence of high quality measurement properties [18]. The CAPL-2 is a refined version of the CAPL [19], which has been used to measure more than 10,000 children’s PL in Canada [20]. CAPL-2 measures four domains of PL: Motivation and Confidence, Physical Competence, Knowledge and Understanding, and Daily Behavior. The tool consists of questionnaires, cognitive and physical tests, and measures of daily behavior [19]. The CAPL was recently the topic of a special issue of *BMC Public Health* where 14 research papers were published showing both how PL measured with CAPL was associated with PA and health-related factors as well as providing evidence of measurement properties of the overall CAPL-2 and the tools and instruments used to measure all four domains individually [21]. Recently, CAPL-2 has been translated into several languages and has been used in research studies from Spain [22], as well as validation studies from China [23] and Greece [24]. Given the widespread use of the CAPL-2 and its demonstrated validity and measurement properties, and the need for a valid and reliable measure for Danish children’s PL, the purpose of this study was to translate the CAPL-2 into Danish language, adapt it to Danish context and to test the measurement properties on a sample of Danish school children eight to 12 years of age.

## Methods

### Recruitment and participants

Participants in this project were Danish school children from 2nd to 6th grade. The participating children were between 8 and 12 years of age (mean = 9.86, SD = 1.47) and 54.3% were female. The children were recruited through cooperation with schools and municipalities across the region of Sealand in Denmark. Efforts to ensure representability of the general Danish population in terms of socioeconomic background and ethnicity were made by including schools from neighborhoods with different proportions of citizens with low socioeconomic status. The schools were recruited by authors PSM and MK by contacting schools directly or through municipalities. The physical education teachers in the schools were offered to have their pupils go through the assessment tool as part of their physical education teaching schedule (corresponding to two modules of physical education classes). In total, 19 schools were contacted of which 14 agreed to participate and two withdrew because of COVID. The final sample included 891 school children from 50 classes in 12 schools.

### Translation and pilot testing

The CAPL-2 questionnaires were translated into Danish language and adapted to the Danish context. For this purpose, a protocol inspired by the World Health Organisation (WHO) protocol for translation and adaptation of instruments [25] was developed. The protocol was divided into four phases.

#### Phase 1: forward translation

Two independent Danish researchers with a degree in sport and exercise science and with Danish as their first language translated the questionnaires as well as the protocols for physical testing. The translated documents were compared and combined into one document by a third independent translator with expertise in sport and exercise science and Danish as a first language.

#### Phase 2: expert panel back-translation

The final translated Danish version was reviewed and back-translated into English by a translator with English as a first language and international expertise in sport and exercise science. This version was compared to the original version by the research group and discrepancies were identified. All members of the research group reviewed the discrepancies and made changes accordingly. The final version was then reviewed by practitioners with experience in teaching physical education and Danish in primary school, to improve understanding of the questions for the age group and further linguistic adjustments were made.

#### Phase 3: pre-testing and cognitive interviewing

The physical tests (i.e. CAMSA, PACER and plank isometric hold; please see section on CAPL-2-related measures later) were pilot tested on 2 second grade classes (the lowest grade included in the sample) at one school. The lowest grade was chosen because it was expected that the youngest children would use the most time filling in the questionnaires as well as doing the physical testing. Therefore, if the practical plan worked in these grades it would be a good indication that it would work in all grades. Hence, based on the pilot in the second grade, solutions to the practical issues of how to administer the different tests within a Danish physical education setting were developed. The questionnaire items were also pilot tested in the same class. The questionnaires were administered, response distributions were inspected, and the following week cognitive interviewing was performed with all children. Here using the lowest grade was also optimal because it was expected that if understandability was achieved in the youngest grade this would also be high in the older classes. At the interviews, the children’s verbal answers were compared to the answers they gave in the questionnaires and they were given the possibility to identify words or concepts they found hard to understand. Based on the feasibility from the pilot, the research group changed the distribution type of the questionnaires, which was originally distributed on paper and administered by a teacher. Hence, the final version of the questionnaire was video-assisted (pictures and audio), so the children could watch and answer on their own on a tablet or computer.

#### Phase 4: final decision making

Finally, the project group went through all previous steps and made decisions on the final translation of the descriptions of the physical tests as well as the questionnaire items. A final version of questionnaire items and manual description was decided upon.

### Measures

#### CAPL-2-related measures

As described briefly in the introduction, CAPL-2 measures PL in four domains: Motivation and Confidence, Physical Competence, Knowledge and Understanding, and Daily Behavior. The domains are measured through a series of questionnaires, cognitive and physical tests, and direct monitoring of daily step counts which when completed are converted into a score ranging from 1 to 100. Detailed descriptions as well as videos with instructions of each protocol for measuring the components within each domain can be found in the CAPL-2 manual, which is available on the CAPL website (https://www.capl-eclp.ca/).

The Motivation and Confidence domain is measured with a 12-item questionnaire. The questionnaire aggregates to four subscales: predilection, adequacy, intrinsic motivation and self-confidence. Each subscale is measured with four items.

Physical Competence is measured with three different physical tests. 1) The plank isometric hold that measures torso muscular endurance [26], 2) the Progressive Aerobic Cardiovascular Endurance Run (PACER), an aerobic fitness test which measures the children’s aerobic capacity [27], and 3) the Canadian Agility and Movement Skill Assessment (CAMSA), which is a dynamic motor skill test developed for children aged 8–12 years of age [28].

The Knowledge and Understanding domain is measured with the Physical Literacy Knowledge Questionnaire (PLKQ) [29], which measures PL-related knowledge, based on the Canadian school curricula. It involves four multiple choice quiz items with four response possibilities. There is one correct and three incorrect answers for each item. Lastly, children complete a comprehension test in form of a ‘fill in the blank’-task where they fill in six blank spaces in a short story about concepts related to PA. All questionnaires are available on the CAPL-2 website.

The Daily Behavior domain is measured with one self-report item of weekly participation in moderate- to vigorous-intensity physical activity and objectively measured average step count for 1 week. Week average of step counts was measured with an AX3 Axivity accelerometer (Ltd., Newcastle upon Tyne, United Kingdom) instead of a pedometer as described in the original CAPL-2 protocol. This was done in order to increase compliance as this method has previously been used with success in a Danish context [30] and accelerometers have been validated in children to measure physical activity, including step counts, with great success [31]. The monitor was affixed on the mid-anterior aspect of the left thigh using a taping method [30], which enable full 24-h recording, since it allows the participant to bath and swim with the monitor attached. Participants were instructed to reinforce the original tape, as required to avoid it from falling off. The measurement period was the week in between the two physical education classes.

#### Physical education teacher rating

To be able to investigate the predictive validity of the Danish version of the CAPL-2, the children’s physical education teachers were asked to rate their pupils on a scale from “1. not true at all” to “10. completely true” on four characteristics, which theoretically should be associated with children’s PL [32]. In all four items, the physical education teachers were asked to rate the child compared to their peers. The four items were (here presented in as direct translation from Danish to English as possible) “*The pupil’s motor skills are generally good*”, “*The pupil seems to enjoy physical education*”, “*the pupil shows a high degree of self-confidence during physical education*” and “*the pupil is skilled in many different types of sport and exercise activities*”. A composite score of the pupil’s total teacher rating was calculated as the average of the four items.

### Procedure

The different measurement protocols of the CAPL-2 were administered during two physical education classes 1 week apart. During the first class, the children completed the CAMSA test, answered the questionnaires of the Motivation and Confidence domain and the cognitive test for the Knowledge and Understanding domain. The second class, the children completed the plank isometric hold and PACER test and answered some additional questionnaires. All physical tests where administered by the research group and trained student research assistants. In between the two classes, the children wore an Axivity AX3 accelerometer continuously. During data collection, the children were divided into three or four groups that alternated between the physical tests and answering the questionnaires and the cognitive tests.

To help the children who had difficulties reading, a video where all items of the questionnaires were read out loud, while the text appeared on the screen, were made. This was embedded in the electronic questionnaire set up in the survey program SurveyXact (Rambøll Management.

Consulting, version 6.10 Copenhagen, Denmark). The participants filled in the questionnaires on a computer or a tablet while wearing headphones. A positive side-effect of wearing headphones was that the participants were quiet and did not interfere with each other while filling in the questionnaires. If necessary, the teacher assisted the children.

#### Ethical considerations

Study procedures were approved by The Capital Region’s center for data reviews “Videnscenter for Dataanmeldelser” (Reference: P-2019-659). In Denmark, only biomedical research and research projects that entail a risk for participants can receive a Trial Registration Number through ethics review by a Regional Ethics Board. The Regional Ethics Board have assessed that the project is not notifiable (journal number:19088122). All methods were carried out in accordance with relevant guidelines and regulations. Written information about the study was given to all school principals, teachers and parents/guardians before the start of the study, and informed consent was obtained from the legal guardians of all participants. The pupils themselves also had the option of withdrawing from the project upon request.

### Data analysis

#### Reliability

To assess internal consistency of psychometric scales, within the Motivation and Confidence domain, robust Cronbach’s alpha was calculated as well as for the entire Motivation and Confidence domain for which the omega reliability measure was also calculated. Reliability measures were computed with the R package ‘coefficientalpha’ [33]. Values above .7 were considered acceptable [34].

#### Construct validity

Construct validity was examined by conducting confirmatory factor analyses. The analyses were done in Mplus [35]. The nested structure of the data was accounted for by specifying the variable school class in the function cluster. Maximum likelihood estimation with robust standard errors (MLR) was used and type was set to complex. The following criteria for an acceptable model fit was used: Chi-square/df < 5.00, comparative fit index (CFI > .95), Tucker-Lewis index (TLI > .95), root mean square error of approximation (RMSEA<.06) and root mean square residual (SRMR<.08) [36]. If an unacceptable model fit was reached, inspection of modification indices informed model fit improvements by either removal of poor indicators or by allowing correlations between error terms of indicators within a domain. Mplus code can be supplied upon request.

#### Predictive validity

The predictive validity of the CAPL-2 was investigated by conducting five regression models with teacher rating of the pupils as outcomes and each of the domains as well as the total CAPL-2 score as predictors. The teacher ratings and PL were hypothesized to be associated, and this association was used as an indication of predictive validity as the indicators that the physical education teachers rated their pupils on have been argued as central characteristics and outcomes of PL [7].

## Results

### Descriptive statistics

Descriptive statistics and correlations between the four different domains and total CAPL-2 score are presented in Table 1. The Motivation and Confidence domain had a high mean of 25.6 given the range of 9–30 indicating a ceiling effect; however, none of the domains had problematic skewness or kurtosis values. Correlations between factors were significant and positive except for the Knowledge and Understanding domain that did not correlate significantly with either the Motivation and Confidence or Daily Behavior.

**Table 1:** Descriptive statistics and correlations between the four domains of the Danish version of CAPL-2

	Descriptives							Correlations				
	N	Mean	SD	min	max	skew	kurt	1	2	3	4	5
1. Motivation and Confidence	799	25.6	4.1	9.0	30.0	−1.1	0.9	1.0				
2. Daily Behavior	609	13.9	5.3	3.0	30.0	0.5	0.1	.28**	1.0			
3. Knowledge and Understanding	771	6.6	2.2	0.0	10.0	−0.6	−0.1	0.00	0.08	1.0		
4. Physical Competence	681	18.6	6.0	2.1	30.0	−0.2	− 0.6	.27**	.28**	.35**	1.0	
5. Total CAPL-2 score	526	65.0	11.8	32.8	97.6	0.1	0.0	.62**	.69**	.41**	.80**	1.0

### Reliability of the psychometric scales

Robust Cronbach alpha values showed good reliability for the four psychometric scales of the Motivation and Confidence domain and were as follows: predilection α = .80, adequacy α = .74, intrinsic motivation α = .86 and perceived confidence α = .84. The entire Motivation and Confidence domain also had good reliability indicators α = .90; Ω = .90.

### Confirmatory factor analysis

Confirmatory factor analysis was done on all pupils with data on at least one of the indicators, which included 891 pupils from 50 different classes.

Initial confirmatory factor analysis revealed a fair (approaching acceptable) model fit. Model fit indices were as follows CFI = .898; TLI = .853; RMSEA = 0.080 (90% CI 0.071–0.090); SRMR = 0.048. Inspection of modification indices revealed that a significant model improvement would be obtained by allowing the error terms between the predilection scale and the adequacy scale within the Motivation and Confidence domain to correlate. This change was made and justified because the two scales are associated theoretically. This change resulted in an acceptable model fit with the following model fit indices CFI = .973; TLI = .957; RMSEA = 0.040 (90% CI 0.033–0.054); SRMR = 0.040.

**Fig. 1:**
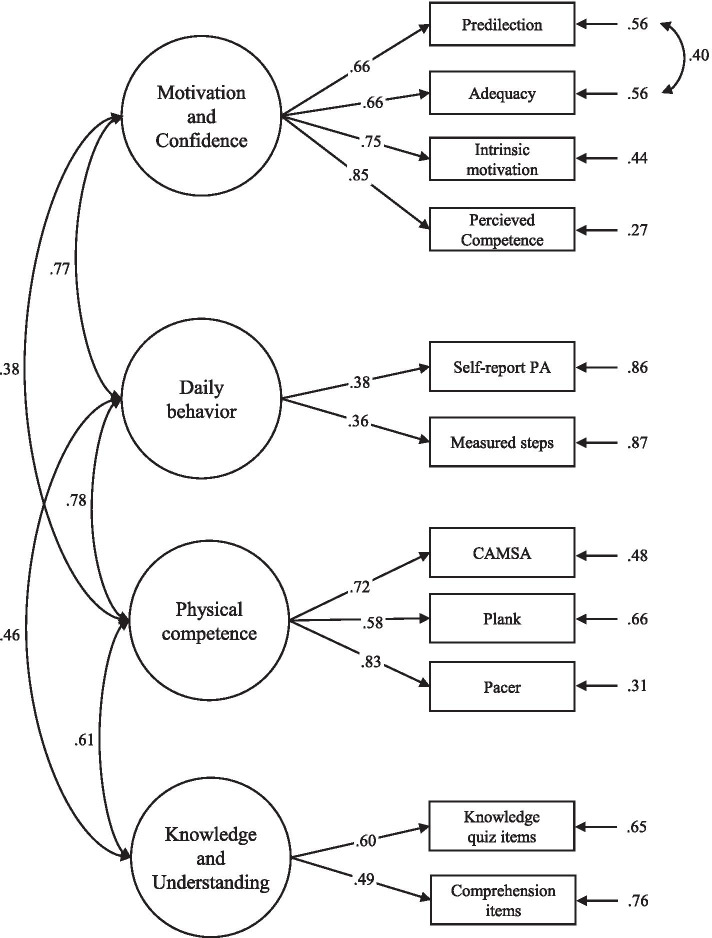
Final confirmatory factor analysis showing significant standardized factor loading, significant factor correlations and significant error term correlations

All factor loadings loaded above .30 (ranging from .36–.85). Factor loadings are illustrated in Fig. 1. Correlations between the latent factors were significant and positive for all factor pairs with the exception of Daily Behavior and Knowledge and Understanding where no correlation was observed (Fig. 1).

### Predictive validity

Five regressions were conducted using each of the domains as well as the total CAPL-2 score as predictors of physical education teacher ratings (Table 2). All domains were significant positive predictors of physical education teacher rating. The strongest single predictor was the Physical Competence domain. Physical Competence explained 21.8% of the variance and showed high predictive power (β = .344). The total CAPL-2 score was an even stronger predictor explaining 31.4% of the variance with high predictive power (β = .560).

**Table 2:** Five regression models showing predictive validity of the Danish version of CAPL-2. The models all have teacher rated PL as outcome and total CAPL score as well as the individual domains as predictors

Predictor	N	B	std er	CI 95 B	β	r^2^	*p*
1. Motivation and Confidence	535	0.166	0.020	.127–.204	0.344	0.118	<.001
2. Daily Behavior	435	0.149	0.016	.117–.180	0.407	0.166	<.001
3. Knowledge and Understanding	520	0.109	0.041	.029–.189	0.116	0.014	0.008
4. Physical Competence	499	0.158	0.013	.131–.184	0.467	0.218	<.001
5. Total CAPL-2 score	390	0.093	0.007	.079–.107	0.560	0.314	<.001

## Discussion

The aim of this study was to translate the Canadian Assessment of Physical Literacy, second edition (CAPL-2) into Danish language, adapt it to Danish context and to test the measurement properties on a sample of Danish school children. Using the four-factor structure as suggested by the original CAPL-2 developers [19, 37], confirmatory factor analysis based on 891 school children, showed that the Danish version of the CAPL-2 fit well to the data. The questionnaires used to measure Motivation and Confidence showed good internal reliability and the overall CAPL-2 score as well as each of the four domains were found to have high predictive validity.

The fit indices and overall indications of validity are in accordance with the evidence suggesting that the CAPL-2 is a valid measure for children aged 8–12’s PL [37]. The fit indices found in this study also align with what has been found in other translation and validation studies from China [23] and Greece [24]. The Danish version of CAPL-2; however, fit well with all indicators of the original CAPL-2 remaining in the model, whereas, in the Chinese version, it was decided to drop the predilection scale as well as one of the Knowledge and Understanding items to reach a better model fit [23]. In the Greek version, all indicators remained in the model; although approaching acceptable, a less optimal fit compared to the Danish version was found. The Greek version also showed problems with low loadings from the predilection scale and three items of the Knowledge and Understanding domain [24].

While the differences in model fit between the different translations of CAPL-2 are relatively small, the obvious difference lies in the different contexts of the samples and the different linguistic translations. That considered, there might be other explanations as well. This study is the first validation study of the CAPL-2 that accounted for the nesting structure of the data. The sample size of this study is substantially larger than the Greek and the Chinese validation study samples. Another difference is that the Knowledge and Understanding domain were modelled with the four multiple choice quiz items as one scale and not as single items as they were in the Canadian, Chinese and Greek evaluations. This was done because of the cultural adaptions made in the Danish version to suit the Danish curricula and the fact that dichotomous variables are discouraged by many researchers when applying confirmatory factor analysis [38]. The approach of helping children’s understanding of the items and the questionnaire as a whole, by video and speech assistance on tablets and headphones might also have improved data quality.

The vast majority of the correlations between the domains found in this study were positive and significant, except from the Knowledge and Understanding domain, which did not correlate significantly with either Motivation and Confidence or Daily Behavior. This pattern completely replicates what was showed in the original validation of the CAPL-2 [37]. The correlation pattern is also almost identical to the Chinese validation study with the exception that a very weak positive significant correlation was found between Knowledge and Understanding and Motivation and Confidence in the Chinese study [23], where no correlation was observed in this study.

This study found indications of predictive validity by showing that both the individual domains and the total CAPL-2-score of the Danish version significantly predicted physical education teacher’s assessment of how much their pupils showed enjoyment, competence, confidence and diversified skill level in physical education classes. These are four areas that are theoretically linked with, and should be associated with PL, meaning that these findings are in accordance with the core of the theoretical concept of PL as first described by Whitehead [7].

In addition to indication of validity, the results found in this study also indicate that the translation and the cultural adaptation phase was successful. This was indicated by the alignment with other versions of CAPL-2 of the found fit indices as well as correlations between the domains. The high proportion of schools and pupils agreeing to participate and remain in the project are also indications of successful cultural adaptation.

### Strengths and limitations

This study has strengths and limitations that need to be considered when interpreting the results. The relatively large sample size, robust data collection by trained researchers and adjustment of the nested data structure are clear strengths of this study. The use of accelerometers to measure children’s steps can be considered a strength as it is a well-established measurement tool for assessing PA over specified time intervals such as 7 days and further a well-validated protocol tested in a Danish sample was used [30]. Another advantage of using our accelerometer protocol compared to using pedometers, is that it meant that the child did not have to remember to put on the pedometer each morning or keep a log of daily step counts which was found in other studies to result missing data [37]. However, it can also be considered a weakness, because it is a different device compared to the one used in the original CAPL-2, which may make comparisons across countries less valid.

The success of the recruitment in this study is indicated by the high percentage of invited pupils that agreed to participate. This is a clear strength as this reduces the likelihood of introducing bias toward one specific group of children. This study used convenient sampling, which is a weakness because this might introduce selection bias. However, efforts were made to account for this weakness as the sample’s distribution on important factors (i.e. the school’s neighborhood average social economic status and the school’s location (rural or urban)) were monitored, considered and guided the selection of schools throughout the data collection phase.

The amount of missing data needs to be considered a limitation. It is notable that 526 of the 891 children that provided consent completed the all protocols to get a full CAPL-2 score. Missing data were mostly due to pupils not attending school on one of the days where measurements were conducted or invalid wear time of the accelerometer. These are reasons that might introduce bias less compared to other typical reasons such as failing to finish a questionnaire because it is too long / difficult or refusing to participate in a physical test. The percentage of missing data was low at the domain level and the data missing for total CAPL-2 was lower compared to the original CAPL-2 validation study [37] and comparable to the two other translation and validation studies [23, 24].

In this study a rating of the pupils by their physical education teachers was used for assessing predictive validity of the Danish version of CAPL-2. This measure has both weaknesses and strengths that need to be considered. The physical education teachers have weekly interactions with their pupils in the physical education context, which means that they have many relevant experiences on which to base their rating. The research-team developed the four questions based on the theory of physical literacy. However, this measure has not previously been validated which is a weakness. This was the first study to translate, adapt and validate a Danish version of CAPL-2. Future research should replicate these results and investigate other types of validity and reliability of the measurement tool such as test-retest reliability of the instruments used and measurement invariance across important background variables such as gender and age.

## Conclusions

This was the first study to translate, adapt and validate a Danish version of CAPL-2, a comprehensive and widely used measurement tool for measuring children’s PL. The results of this study underline the usefulness of CAPL-2 and provide evidence that the Danish version achieved factorial validity. The instruments used showed high internal consistency and indications of predictive validity of each domain and total CAPL-2 score, with the overall conclusion that the translated and context adapted Danish version of CAPL-2 is a valid and reliable measurement tool. With this valid and reliable Danish PL assessment tool, intervention-based PA research in Denmark now has a comprehensive, relevant and meaningful outcome measure for evaluating and guiding interventions and initiatives aiming to promote children’s PL and thereby lifelong PA engagement. The measurement tool not only serves the purpose of informing existing interventions’ effect, but also provides directions to future interventions that aim to improve the Danish population’s activity habits.

## Data Availability

The datasets used and/or analysed during the current study is available from the corresponding author on reasonable request.

## Abbreviations

CFI: Comparative fit index
TLI: Tucker-Lewis index
RMSEA: Root mean square error of approximation
SRMR: Root mean square residual
CAPL: Canadian Assessment of Physical Literacy
CAPL-2: Canadian Assessment of Physical Literacy second edition
PA: Physical Activity
WHO: World Health Organisation
CAMSA: Canadian Agility and Movement Skill Assessment
PLKQ: Physical Literacy Knowledge Questionnaire
PACER: Progressive Aerobic Cardiovascular Endurance Run
